# Internal-Control-Normalized T2 Mapping of Meniscal Abnormalities on a Cartilage MRI Protocol: An Exploratory Retrospective Study

**DOI:** 10.3390/diagnostics16162564

**Published:** 2026-08-14

**Authors:** Wenwen Ni, Mun Sze Khaw, Phey Ming Yeap, Mohammed Taufik Bin Mohamed Shah, Cynthia Assimta Peter, Jamie Xiu Mei Ho, Yi Xian Low, Dickson Hong Him Chau, Jeremy Keng Meng Goh, Steven Bak Siew Wong, Adriel Guang Wei Goh

**Affiliations:** 1 Department of Radiology, Sengkang General Hospital, Singapore 544886, Singapore; wenwen.ni@mohh.com.sg (W.N.); khaw.mun.sze@singhealth.com.sg (M.S.K.); yeap.phey.ming@singhealth.com.sg (P.M.Y.); mohd.taufik.mohd.shah@singhealth.com.sg (M.T.B.M.S.); cynthia.assimta.peter@singhealth.com.sg (C.A.P.); jamie.ho.x.m@skh.com.sg (J.X.M.H.); low.yi.xian@skh.com.sg (Y.X.L.); steven.wong@singhealth.com.sg (S.B.S.W.); 2 Department of Orthopaedic Surgery, Sengkang General Hospital, Singapore 544886, Singapore; dickson.chau.h.h@singhealth.com.sg (D.H.H.C.); jeremy.goh.k.m@singhealth.com.sg (J.K.M.G.)

**Keywords:** T2 relaxation value, quantitative MRI, cartilage, meniscal tear, meniscal degeneration, meniscal pathology, arthroscopic correlation

## Abstract

**Background/Objectives**: Meniscal pathology is a common consequence of knee injury and a precursor of osteoarthritis, but qualitative MRI grading cannot reliably distinguish low-grade intrasubstance degeneration from more advanced pathology. Quantitative T2 relaxation value (T2RV) mapping is an established cartilage marker, but internal-control normalisation within the same meniscus has not been formalised for incidental meniscal pathology. We hypothesised that pathologic meniscal regions would show elevated T2RV relative to internal-control tissue. **Methods**: In this retrospective, single-centre study, 61 pathologic meniscal lesions with arthroscopic correlation (12 tears, 40 repaired, 9 myxoid/intrasubstance degeneration) in 55 patients (mean age 47.1 ± 8.8 years) underwent a cartilage T2RV MRI protocol. A pathologic and a remote internal-control region of interest (ROI) were placed on the T2RV colour map for each lesion; the T2RV endpoint was (pathologic − internal-control ROI T2RV)/internal-control ROI T2RV × 100%. Groups were compared using Wilcoxon signed-rank and Kruskal–Wallis (Dunn’s post hoc) tests and multivariable linear regression. **Results**: Pathologic ROI T2RV exceeded internal-control ROI T2RV (*p* < 0.001; mean elevation 55.7%). The endpoint varied among pathology groups (*p* < 0.001): degeneration showed the smallest elevation (17.5%), while repair (60.0%) and tear (69.9%) were similarly higher and indistinguishable, remaining independently associated with a higher endpoint than degeneration after multivariable adjustment. Longer postoperative intervals were associated with a lower endpoint among repaired lesions (β = −6.27 points/100 days). **Conclusions**: Internal-control-normalised T2RV mapping is a promising quantitative marker of meniscal tissue status that may complement conventional MRI and clinical assessment, with T2RV elevation differing among myxoid/intrasubstance degeneration, tear, and repaired meniscus within this cohort. Prospective, multicenter validation is warranted before clinical adoption.

## 1. Introduction

The meniscus is a key load-bearing, shock-absorbing, and stabilising structure of the knee, and meniscal injury is among the most common injuries of this joint, with a rising annual incidence [1]. Meniscal injury is not a self-limiting problem. On average, 50% of patients with a diagnosed anterior cruciate ligament (ACL) or meniscus tear develop osteoarthritis, with associated pain and functional impairment, within 10 to 20 years of diagnosis—“the young patient with an old knee” [2]. Traumatic tears, occurring in a previously healthy meniscus, and degenerative tears, arising from repetitive loading on an already-worn meniscus, differ in typical patient age, tear morphology, and prognosis, but both are linked to long-term joint degeneration [1,2].

A degenerative tear—caused by wear rather than trauma—is, by definition, still a tear, with signal reaching the articular surface (Grade III); myxoid/intrasubstance degeneration, in contrast, denotes abnormal signal on MRI confined within the meniscus (Grade I/II) [3]. In a randomised, sham-controlled trial of arthroscopic partial meniscectomy for degenerative meniscal tears without osteoarthritis, outcomes at 12 months did not differ between patients who underwent surgery and those who underwent a sham procedure [4]. This finding has reshaped how degenerative tears are managed and underscores that not every MRI-detected meniscal abnormality represents a lesion that could benefit from intervention.

However, in practice, this Grade I/II-versus-Grade-III distinction is imperfect. Intrasubstance signal on MRI has been shown to correspond to a true, arthroscopically confirmed tear in a minority of cases, and true-tear-pattern signal has been reported in roughly half of asymptomatic patients over 40 years of age with osteoarthritis [3]. Isolated intrasubstance meniscal degeneration is also common and often follows a benign, largely asymptomatic course, though a subset progresses to true tear with risk factors such as weight gain [5]. Meniscal pathology—whether tear or intrasubstance degeneration—is therefore frequently incidental, and conventional MRI grading alone cannot reliably separate clinically inert degeneration from a lesion that reflects, or predisposes to, more advanced joint disease. Postoperative MRI assessment of the meniscus remains similarly challenging, particularly years after repair [6,7,8], underscoring a broader need for a quantitative, rather than purely qualitative, approach to meniscal tissue characterisation.

Quantitative relaxometry has emerged as an alternative that moves beyond binary signal-grade assessment. Several technologies, e.g., ultrashort-echo-time T2*, synthetic-MRI-derived T2, radial T2* mapping, and ultrahigh-field T2* mapping, have been shown to detect subclinical meniscal injury invisible to conventional imaging and arthroscopy. In these studies, quantitative values track tissue change and, longitudinally, normalise over time in intact but not torn menisci [9,10,11,12,13,14,15,16]. However, most of these studies report meniscal relaxation values either as raw, absolute measurements or against an external healthy-volunteer reference. The cartilage literature has addressed an analogous problem by expressing repair-tissue relaxation as an index relative to a reference region of native tissue in the same joint [17]. Quantitative T2-relaxation value (“T2RV”) assessmentrelative to adjacent native tissue is already a standard interpretive practice for postoperative cartilage grafts [18,19]. However, a cartilage-oriented T2 protocol may not be fully optimised for the shorter native T2 relaxation characteristics of meniscal fibrocartilage relative to hyaline cartilage and may be affected by partial-volume and orientation-related effects.

The present study used a clinically established cartilage T2RV colour mapping protocol to evaluate a retrospective, single-centre cohort with incidentally identified meniscal pathology (tear, repaired meniscus, or myxoid/intrasubstance degeneration). We hypothesised that pathologic regions would show elevated T2RV relative to normal tissue, with this elevation differing across pathology types. Specifically, we hypothesised that internal-control-normalised T2RV would differ between myxoid/intrasubstance degeneration and structural tear and repair, reflecting differences in underlying tissue composition and biological state.

## 2. Materials and Methods

### 2.1. Study Design and Population

**Figure 1:**
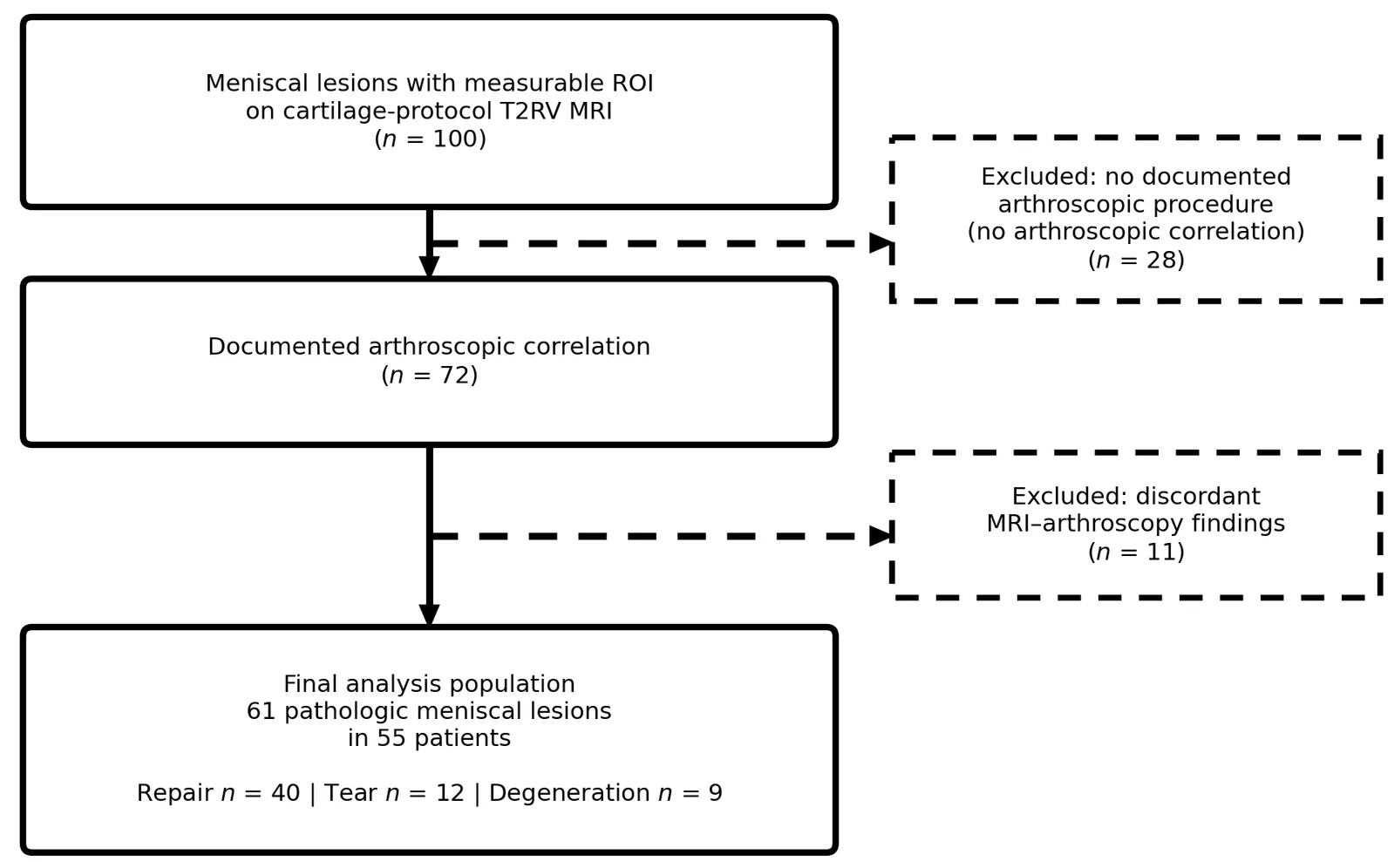
Patient/lesion selection flow diagram. Of the 100 lesions with adequate image quality for ROI measurement, 28 were excluded for absence of documented arthroscopic correlation, and 11 were further excluded for discordant MRI report and arthroscopic findings, yielding the final analysis population of 61 lesions.

This retrospective, single-centre study was conducted at a national tertiary hospital and approved by the SingHealth Centralised Institutional Review Board (CIRB) (protocol code 2020/2381). IRB approval has been maintained through continuous renewal for the duration of the study: approval was valid through 6 November 2025 per the renewal reviewed on 7 November 2024 (Study Status Report Form Version 1, dated 5 November 2024), and the most recent renewal, granted 10 September 2025, is valid through 9 September 2026 (Study Status Report Form Version 2, dated 3 September 2025); the requirement for informed consent was waived. The population comprised patients who underwent knee MRI with a cartilage protocol including T2 relaxation value (T2RV) colour mapping for preoperative assessment of chondral injury or postoperative surveillance after cartilage grafting or repair, performed between 15 April 2021 and 6 October 2025. Patients with incidental meniscal pathology visible on this MRI protocol—meniscal tear, myxoid/intrasubstance degeneration, or repaired meniscus—and adequate image quality for region o interest (ROI) measurement were included, and arthroscopic correlation was required for inclusion in the final analysis population, applied uniformly as the reference standard for lesion confirmation. Cases were excluded for inadequate image quality precluding ROI measurement (motion, susceptibility, or postoperative artefact; missing or technically inadequate T2RV sequence), absent internal-control tissue (extensive meniscal destruction, maceration, or prior resection with no remote normal-appearing region available), discordance between the MRI report and arthroscopic findings (i.e., a tear reported on MRI but a normal meniscus at arthroscopy), or absence of a documented arthroscopic procedure at the time of this analysis. Of the 100 lesions with adequate image quality for ROI measurement, 28 were excluded for absence of documented arthroscopic correlation, and 11 were further excluded for discordant MRI report and arthroscopic findings, yielding the final analysis population of 61 lesions (Figure 1). Where a patient had more than one MRI study relative to a given lesion, only the single study closest in time to the arthroscopic procedure—whether performed before or after surgery—was selected for analysis.

The final analysis population comprised 61 pathologic meniscal lesions with arthroscopic correlation in 55 patients. The unit of analysis was the individual pathologic meniscal lesion; patients with pathology involving more than one meniscus, or with both a preoperative and a postoperative lesion, contributed one observation for each affected meniscus. 6 patients (10.9%) contributed more than one lesion, accounting for 12 of the 61 lesions (19.7%); a sensitivity analysis retaining a single lesion per patient is reported in the Supplementary Materials (Supplementary Table S1).

### 2.2. Image Acquisition

MRI was performed using a 3T MRI unit (Signa Architect; GE Healthcare, Waukesha, WI, USA) with an 18-channel transmit and receive knee coil, using AIR Recon DL (ARDL) technology. Both multiplanar conventional MRI sequences and T2RV maps of the cartilage were obtained. Conventional sequences include coronal T1 fast-spin echo (FSE), coronal proton density (PD) FSE with fat saturation (FS), sagittal T2 FSE FS, sagittal PD FSE 1.5 mm, and axial PD FSE FS. Coronal, sagittal, and axial T2RV mapping were performed with a 130 mm field of view, pixel size of 0.5 × 0.5 mm, slice thickness of 3 mm, repetition time of 1200 ms, and 8 variable echo times. The 8 echo times were 7.1, 14.2, 21.3, 28.4, 35.6, 42.7, 49.8, and 56.9 ms in the sagittal plane and 7.8, 15.6, 23.5, 31.3, 39.1, 46.9, 54.8, and 62.6 ms in the axial plane, with the coronal plane using the same acquisition scheme. Reconstructed matrices were zero-padded in k-space to the next power of two on DICOM output (i.e., 140 × 140 interpolated to 256 × 256 and 280 × 280 interpolated to 512 × 512). The T2RV maps were generated using the T2RV map algorithm from Cartigram (GE Healthcare, Waukesha, WI, USA), which computes T2RV relaxation time in milliseconds via a pixel-wise least-squares linear fit of the log-transformed signal intensity against echo time, the standard linearisation of the mono-exponential T2 decay model. The fit was performed after first discarding trailing echoes whose signal intensity fell below a user-defined noise threshold (default 20), and the first echo was included by default. Fit quality was governed by comparison of the resultant chi-squared value against a reference threshold derived from a user-specified confidence level (default 0.05), and a minimum–maximum clipping operator additionally excluded values outside a range of 0–80 ms from the colour map. T2RV values are illustrated using a colour-coded map, with shades of purple, blue, green, yellow, and red, represented on a colour-coded scale with a lower limit of 0 ms and an upper limit of 80 ms. In the present study, this T2RV mapping and colour-coded approach, originally established for cartilage, was applied using the same acquisition parameters to the meniscus.

### 2.3. Meniscal Pathology Assessment and ROI Measurement

Conventional morphological MRI sequences and quantitative T2RV colour maps were each independently reviewed by a fellowship-trained musculoskeletal radiologist. The radiologist placing the ROIs was blinded to arthroscopy, surgery date, and clinical history.

Incidental meniscal pathology was categorised as tear, myxoid/intrasubstance degeneration, or repaired meniscus. Myxoid/intrasubstance degeneration was defined as globular or linear intrasubstance signal hyperintensity on conventional sequences that did not extend to an articular (superior or inferior) surface of the meniscus; signal extending to an articular surface was defined as a tear. For tears, morphology was additionally recorded as horizontal, vertical, radial, root-related, complex, or bucket-handle; root-related tears were defined as radial tears located within 1 cm of the native meniscal root attachment. Meniscal side (medial or lateral) and region (anterior horn, body, posterior horn, anterior root, or posterior root) were recorded for each lesion. Lesion pathology type and location were solely identified from conventional morphological MRI. Region of interest (ROI) measurements were performed on CartiGram software using a standard circular-shaped ROI marker. The ROI measurement tool was set to its fixed smallest possible size of 5 pixels^2^, corresponding to approximately 0.1 mm^2^. A single pathologic ROI was placed on the T2RV colour map at the site of the tear, degeneration, or repair; when a tear extended across more than one region, the ROI was placed on the most visually abnormal measurable portion of the lesion on combined review of conventional morphology and the T2RV map. A separate internal-control ROI was placed within a remote, morphologically normal-appearing region of the same meniscus; in patients with pathology affecting more than one meniscus, an independent internal-control ROI was measured within each affected meniscus. Where available, arthroscopy served as the reference standard for tear confirmation and repair status. Arthroscopic procedures were performed by one of seven fellowship-trained orthopaedic surgeons, with 6–13 years of specialist experience, using the ISAKOS classification system.

### 2.4. Primary Endpoint

The primary endpoint was defined for each lesion as the relative difference between the pathologic ROI and the internal-control ROI, normalised to the internal-control ROI:

T2RV endpoint = (Pathologic ROI T2RV − Internal-control ROI T2RV)/Internal-control ROI T2RV × 100%

The endpoint is reported as a percentage relative elevation over the patient’s own internal-control tissue. This internally normalised endpoint was used in preference to the absolute pathologic T2RV value because it references each lesion to the patient’s own remote normal-appearing tissue, reducing the influence of inter-subject and scanner-related variability in absolute T2RV.

### 2.5. Statistical Analysis

Clinical and imaging data were recorded in Microsoft Excel (Microsoft Corporation, Redmond, WA, USA). Continuous variables are presented as mean ± standard deviation or median (interquartile range), as appropriate; categorical variables are summarised as counts and percentages. Comparisons between paired pathologic and internal-control ROI T2RV values were performed using the Wilcoxon signed-rank test. Comparisons of the normalised T2RV endpoint among pathology groups (tear, degeneration, repair) were performed using the Kruskal–Wallis test, with Dunn’s post hoc test and Holm correction for pairwise comparisons; exploratory comparisons of the endpoint by meniscal side, region, and tear morphology were also performed using the Kruskal–Wallis test. Associations between continuous variables were assessed using simple linear regression/Pearson correlation, with Spearman correlation performed as a non-parametric sensitivity analysis. Multivariable linear regression of the endpoint on sex, age, meniscal pathology group, side and region was performed, with heteroscedasticity-robust (HC3) and, given that six patients contributed more than one lesion, patient-cluster-robust standard errors, to identify independent predictors of the endpoint. A parametric equivalent of the primary comparison (paired *t*-test), concordant with the non-parametric result, is reported in the Supplementary Materials (Supplementary Table S2). Statistical analyses were performed using Python version 3.11 (Python Software Foundation, Wilmington, DE, USA; pandas and SciPy libraries). A generative AI system (Claude Sonnet 5, Anthropic) was additionally used for cross-checking statistical analysis code against the raw dataset, as detailed in the Acknowledgements. A two-sided *p*-value < 0.05 was considered statistically significant.

## 3. Results

### 3.1. Patient and Lesion Characteristics

A total of 61 pathologic meniscal lesions with arthroscopic correlation were analysed in 55 patients (34 men, 21 women; mean age 47.1 ± 8.8 years, range 24–66). Six patients (10.9%) contributed more than one lesion, accounting for 12 of the 61 lesions (19.7%). Lesions involved the medial meniscus in 39 cases (63.9%) and the lateral meniscus in 22 cases (36.1%). By region, the posterior horn was most frequently affected (*n* = 27, 44.3%), followed by the body (*n* = 16, 26.2%), anterior horn (*n* = 12, 19.7%), and posterior root (*n* = 6, 9.8%); no anterior-root lesions retained arthroscopic correlation, so this region is absent from the present cohort.

Lesions comprised repair (*n* = 40, 65.6%), tear (*n* = 12, 19.7%), and myxoid/intrasubstance degeneration (*n* = 9, 14.8%); requiring arthroscopic correlation preferentially retained repaired and degeneration lesions, which are each identified around a surgical event, while excluding most preoperative tears not yet taken to surgery (Section 5, Limitations). Among the 52 tear and repair lesions with tear morphology recorded, complex tears were most common (*n* = 19, 36.5%), followed by radial (*n* = 9, 17.3%), horizontal (*n* = 9, 17.3%), vertical (*n* = 7, 13.5%), bucket-handle (*n* = 5, 9.6%), and root-related (*n* = 3, 5.8%) tears. Among the 42 lesions with a recorded intraoperative status, surgical repair was performed in 23 (54.8%), debridement in 9(21.4%), meniscectomy in 8(19.0%), and 2(4.8%) required no intraoperative intervention despite a documented lesion. MRI indication was postoperative surveillance in 51 examinations (83.6%) and preoperative in 10 (16.4%), recorded descriptively per the study design (Section 2.1) rather than as a comparative variable. Full patient demographics and lesion characteristics are summarised in Table 1.

### 3.2. Representative Imaging Findings

**Figure 2:**
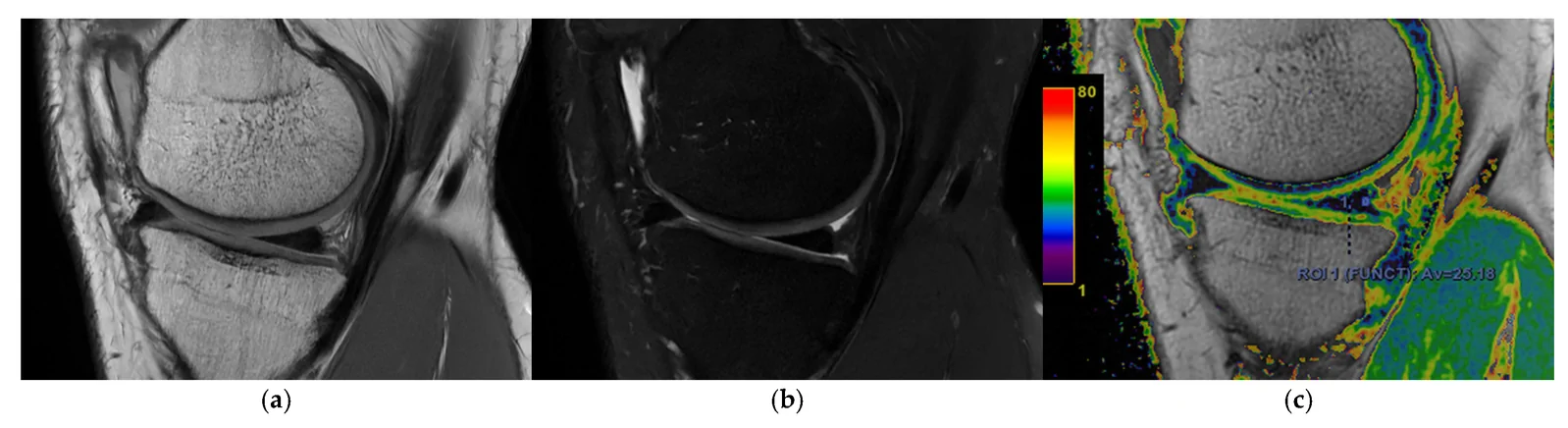
Representative normal medial meniscus. (**a**) Proton density (PD) sequence; (**b**) T2 fat saturation (FS) sequence; (**c**) T2 relaxation value (T2RV) colour map, showing a homogeneously low T2RV without focal elevation (internal-control ROI T2RV 25.18 ms).

**Figure 3:**
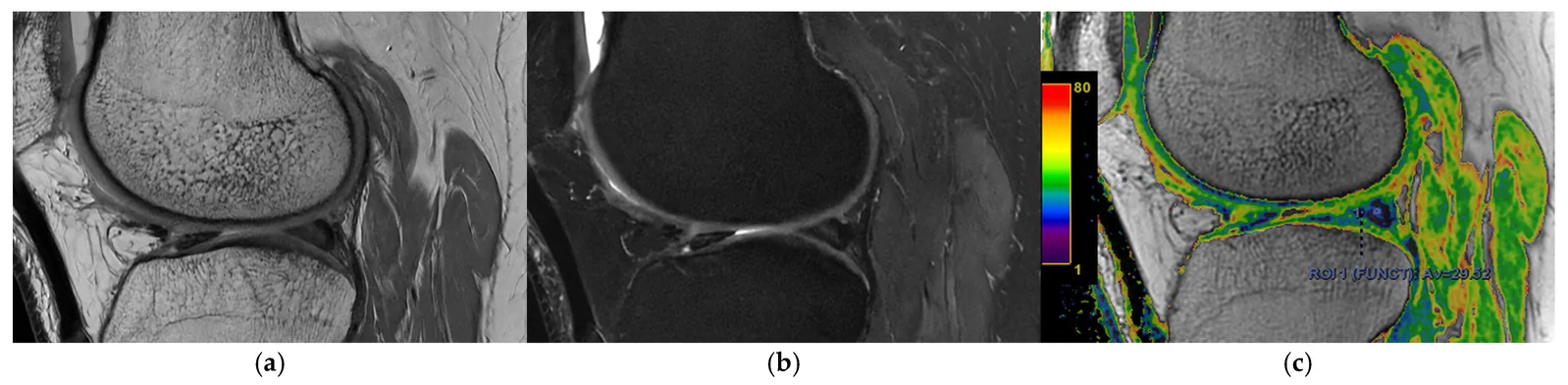
Representative myxoid/intrasubstance degeneration of the lateral meniscus (posterior horn). (**a**) PD sequence; (**b**) T2 FS sequence; (**c**) T2RV colour map, showing a diffuse, mild elevation in T2RV without a discrete tear reaching the articular surface (pathologic ROI T2RV 29.52 ms vs. internal-control ROI T2RV 25.46 ms; T2RV endpoint 15.9%).

**Figure 4:**
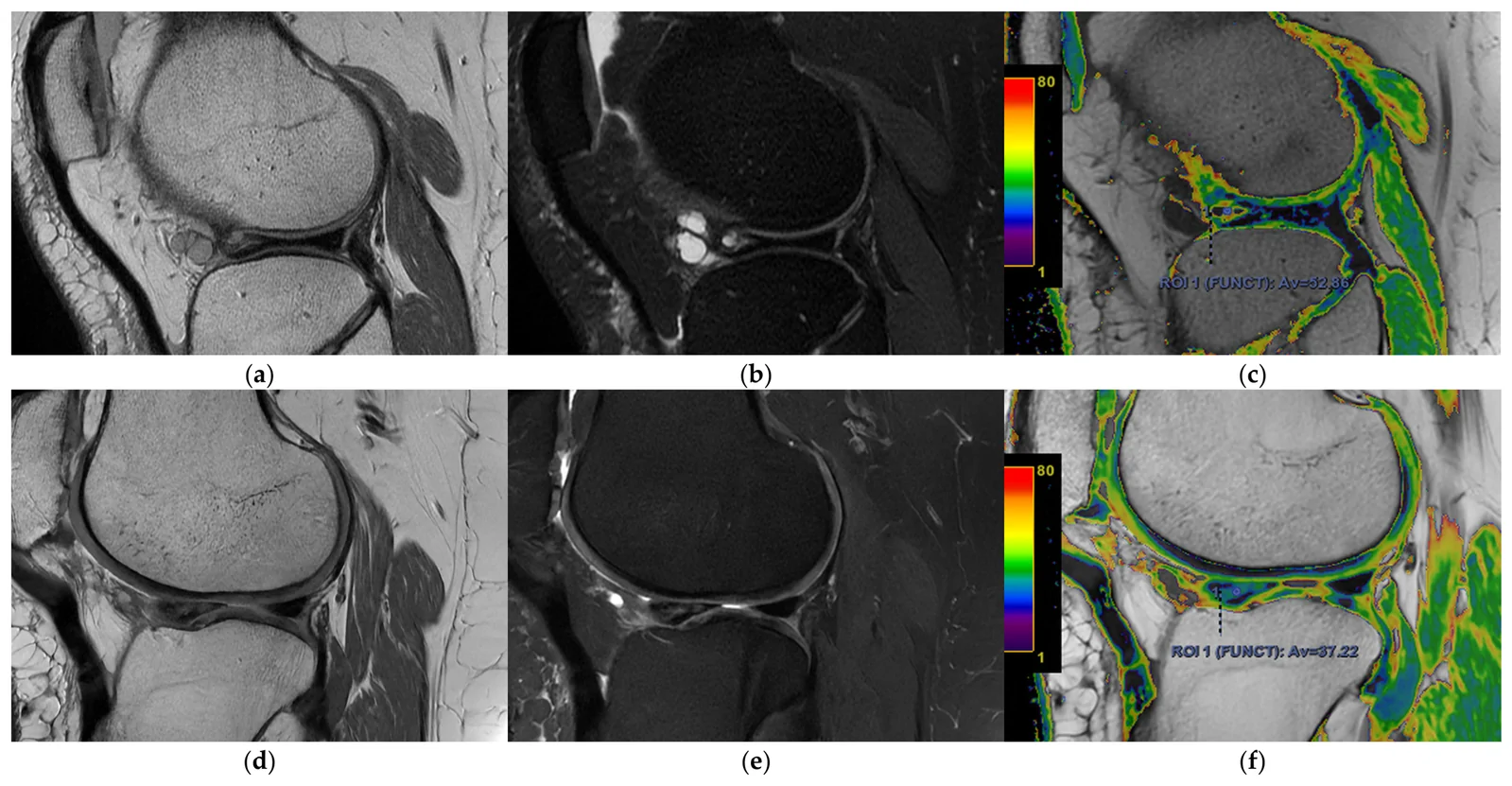
Representative torn meniscus before and after arthroscopic repair in the same patient (lateral meniscus, anterior horn, horizontal tear morphology). **Top row**, before arthroscopic repair: (**a**) PD sequence; (**b**) T2 FS sequence; (**c**) T2RV colour map, showing elevated T2RV at the tear with associated multiloculated parameniscal cyst (pathologic ROI T2RV 52.86 ms vs. internal-control ROI T2RV 26.26 ms; T2RV endpoint 101%). **Bottom row**, after arthroscopic repair: (**d**) PD sequence; (**e**) T2 FS sequence; (**f**) T2RV colour map, showing a reduction in T2RV at the repair site relative to the preoperative study, with the parameniscal cyst mostly decompressed after surgery (pathologic ROI T2RV 37.22 ms vs. internal-control ROI T2RV 25.00 ms; T2RV endpoint 48.9%).

Representative proton density (PD), T2, and T2 relaxation value (T2RV) colour map images are shown for a normal meniscus (Figure 2), myxoid/intrasubstance degeneration (Figure 3), and a torn meniscus before and after arthroscopic repair in the same patient (Figure 4).

### 3.3. Primary Analysis: Pathologic Versus Internal-Control ROI T2RV

Pathologic meniscal tissue demonstrated significantly higher quantitative T2RV than internal normal-appearing meniscal tissue (Wilcoxon signed-rank test, *p* = 1.1 × 10^−11^); a concordant paired *t*-test gave the same conclusion and is reported in the Supplementary Materials (Supplementary Table S2). Pathologic ROI T2RV (mean 40.15 ± 8.89 ms) were consistently higher than paired internal-control ROI T2RV (mean 25.76 ± 0.56 ms) in the same meniscus, with a mean paired difference of 14.39 ± 8.70 ms (95% CI 12.16 to 16.62 ms). The mean normalised T2RV endpoint was 0.557, corresponding to a 55.7% increase in T2RV relative to the internal-control ROI (median 0.562 [56.2%], IQR 0.272–0.712 [27.2–71.2%]) across all 61 lesions. Full results are shown in Table 2.

### 3.4. T2RV Endpoint by Pathology Group

The normalised endpoint differed significantly among the tear, degeneration, and repair groups (Kruskal–Wallis test, *p* = 6.2 × 10^−4^). Post hoc Dunn’s tests with Holm correction demonstrated significantly higher endpoints in tear and repair lesions compared with degeneration (degeneration vs. repair *p* = 1.1 × 10^−3^, degeneration vs. tear *p* = 1.1 × 10^−3^), whereas no significant difference was observed between tear and repair lesions (*p* = 0.400). The degeneration group showed the lowest endpoint (mean 17.5 ± 15.7%, median 13.4%), while the tear (mean 69.9 ± 43.6%, median 79.6%) and repair (mean 60.0 ± 26.1%, median 57.3%) groups showed substantially higher and similar endpoints. This pathology-group separation persists after multivariable adjustment (Section 3.6).

**Figure 5:**
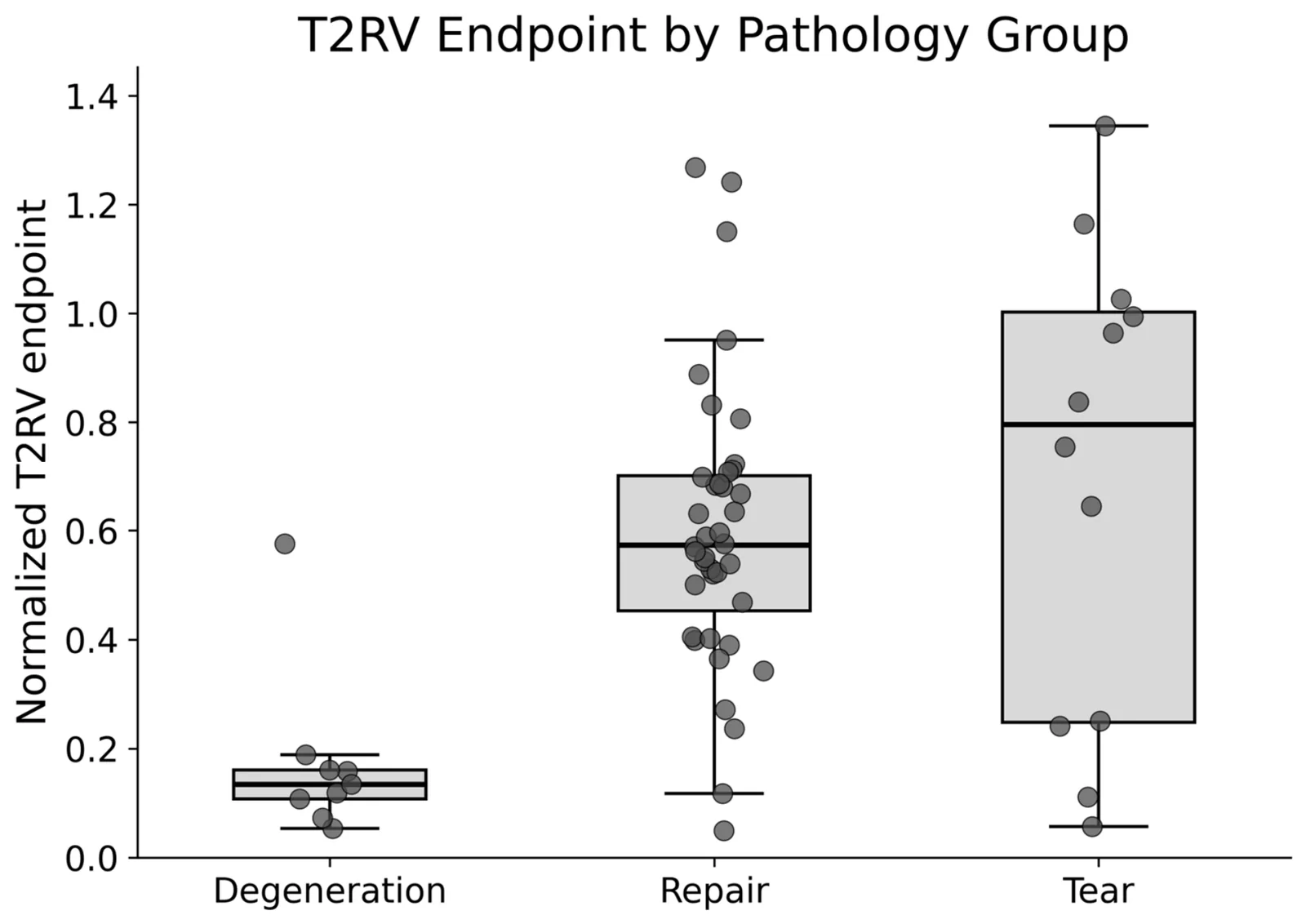
Normalised T2RV endpoint by pathology group. Box plots show the median (horizontal line) and interquartile range (box) for each pathology group; whiskers extend to the most extreme value within 1.5× the interquartile range of the box edges, the standard Tukey convention. Individual lesion values, including any beyond the whiskers, are overlaid as jittered points.

Each pathology group’s own within-group paired comparison (pathologic ROI vs. its internal control) was independently significant, including degeneration despite its small size (*n* = 9; exact Wilcoxon *p* = 3.9 × 10^−3^). This confirms that myxoid/intrasubstance degeneration showed reliably detectable elevation over internal-control tissue, at roughly a third the magnitude of tear or repair lesions. Summarised results are shown in Table 3 and Figure 5, with the within-group paired-test breakdown provided in Supplementary Material (Supplementary Table S3).

### 3.5. Exploratory Analyses by Meniscal Side, Region, and Tear Morphology

The T2RV endpoint did not differ significantly by meniscal side (Kruskal–Wallis *p* = 0.730), by region (Kruskal–Wallis *p* = 0.0503), or by tear morphology among tear and repair lesions (Kruskal–Wallis *p* = 0.139). The region comparison did not reach the conventional threshold for statistical significance, with the posterior horn showing the lowest median endpoint (39.9%), while the posterior root and body showed the highest median endpoints (70.4% and 65.7% respectively). Tear morphology demonstrated numerical variation in normalised endpoint, with root-related and vertical tears showing the highest median endpoints (72.3% and 71.2%), though it does not reach statistical significance. Full subgroup exploratory analyses are provided in Supplementary Table S4.

### 3.6. Multivariable Regression Analysis

Multivariable regression analysis demonstrated that pathology group remained an independent predictor of normalised T2RV elevation, whereas age, sex, meniscal side, and lesion location were not independently associated. Relative to degeneration, both repair and tear were associated with higher normalised T2RV elevation after adjustment (repair *p* < 0.001; tear *p* = 0.011), confirming the group-level difference observed in Section 3.4. Conclusions remain directionally unchanged whether standard errors were adjusted for heteroscedasticity alone (HC3) or additionally clustered by patient to account for the 6patients contributing multiple lesions. Full regression coefficients, together with 95% confidence intervals, are provided in Supplementary Table S5.

### 3.7. Postoperative Interval and T2RV Endpoint

**Figure 6:**
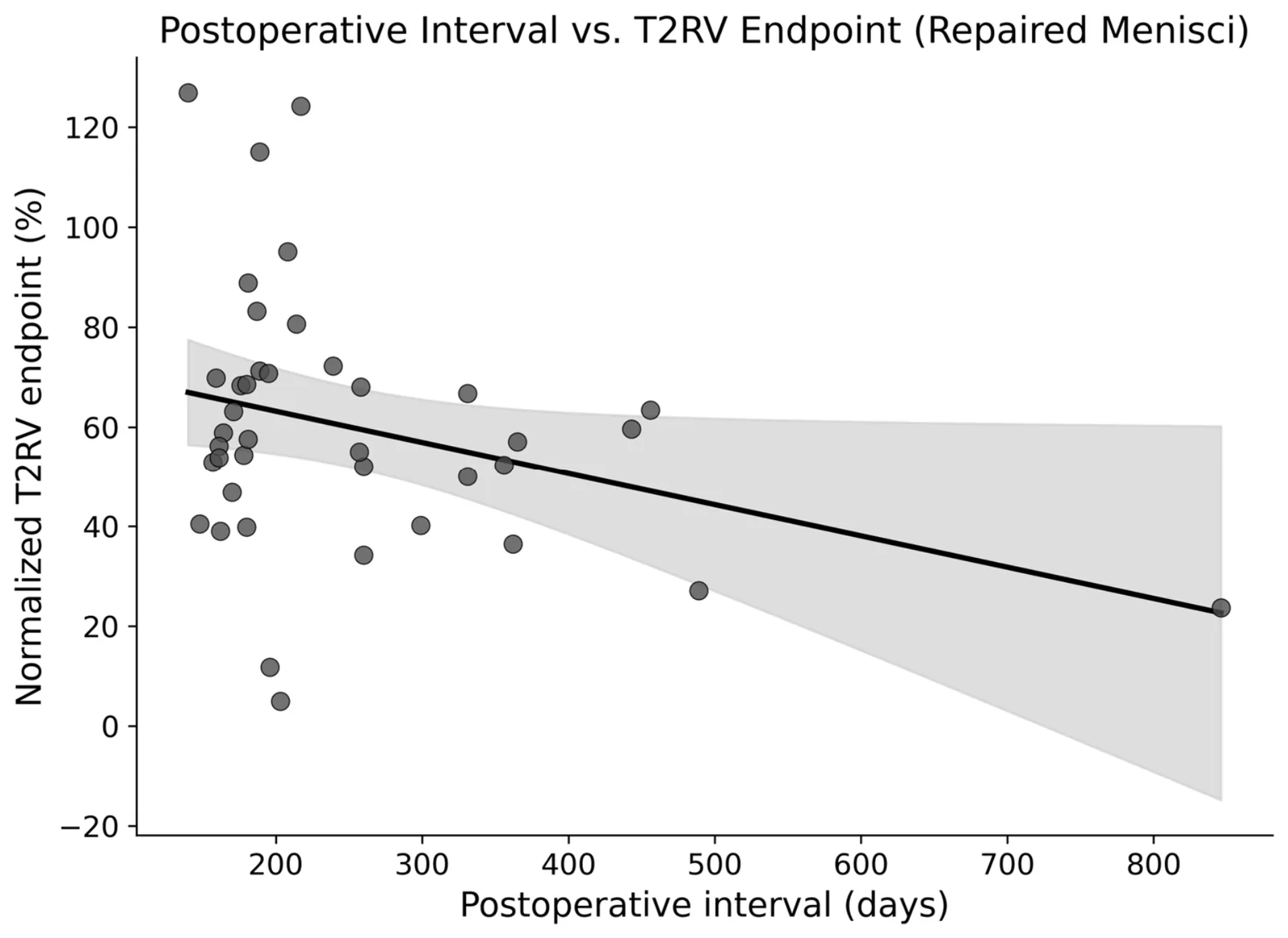
Relationship between postoperative interval and normalised T2RV endpoint in repaired menisci (*n* = 40). Each point represents one repaired meniscal lesion. The solid line represents the fitted linear regression, with the shaded area indicating the 95% confidence interval. A weak inverse association was observed (β = −6.27 percentage points per 100 days).

Among the 40 repair lesions with a recorded postoperative interval (mean 250 ± 132 days, range 140–846 days), simple linear regression showed that a longer interval was associated with a lower T2RV endpoint (β = −6.27 percentage points per 100 days, 95% CI −12.42 to −0.12; *p* = 0.046; Spearman ρ = −0.20, *p* = 0.21) (Figure 6). As a further sensitivity analysis, a full leave-one-out analysis was performed. The direction of the association remained negative after excluding the most influential lesion (longest postoperative interval, 846 days; β = −6.46 percentage points per 100 days), though statistical significance was not reached (95% CI −15.59 to 2.67; *p* = 0.16; Spearman ρ = −0.15, *p* = 0.36). These results suggest a weak inverse association in this cross-sectional sample and should be interpreted as exploratory rather than as direct evidence of temporal healing.

## 4. Discussion

This study evaluated an internal-control-normalised T2RV endpoint across 61 incidentally identified, arthroscopically correlated meniscal lesions and found elevation of pathologic regions (mean normalised elevation 55.7%; *p* < 0.001). The elevation varied by pathology type: myxoid/intrasubstance degeneration showed the smallest elevation (mean 17.5%), while repaired meniscus and tear were higher, at 60.0% and 69.9%, respectively. Together, these findings support T2RV as a potential quantitative marker of meniscal tissue status.

T2RV elevation in pathologic meniscal lesions is consistent with established mechanisms of meniscal tissue disruption. Early degenerative change in fibrocartilaginous tissue involves proteoglycan loss and collagen network deterioration, which increases water mobility and content [20]. This mechanism can be applied to the meniscus, not just cartilage, since T2 relaxation is inherently sensitive to water-proton mobility within the collagen–proteoglycan matrix—a relationship validated in large cartilage cohorts [21,22] and in direct comparison with T1rho-based mapping [23]. Meniscal T2 elevation correlates strongly with histopathological degeneration scores in ex vivo tissue, particularly loss of surface integrity and collagen disorganisation [12,24]. The degeneration group’s comparatively modest elevation may reflect an earlier, less disrupted stage of tissue change than frank tear or repair.

Isolated intrasubstance degeneration may not be fully represented at arthroscopy, since inspection assesses the meniscal surface rather than internal composition. This was reflected in our own degeneration group: an explicit intraoperative description of degeneration was documented in only 1 of 9 lesions (patient code 46), with the remaining operative notes recording an intact-appearing meniscus or, where a co-existing tear was present elsewhere, only the tear. Two explanations are plausible and not mutually exclusive. First, myxoid/intrasubstance degeneration by definition does not extend to an articular surface and need not produce a visible or probe-detectable abnormality; intrasubstance MRI signal corresponds to a true, arthroscopically confirmed tear in only a minority of cases [3], consistent with the earlier-stage change already noted above. Second, operative documentation is procedure- and finding-oriented, with no standard descriptor corresponding to the radiological term “degeneration”; a meniscus without a discrete tear may be conventionally recorded simply as intact, although documentation practices likely differ among arthroscopists. A comparable disconnect between compositional MRI and gross intraoperative impression has been documented in the reverse direction at our own institution, where a postoperative cartilage graft with reassuring MRI was nonetheless non-viable fibrocartilage at arthroscopy [25]. The absence of an explicit operative descriptor should therefore not, by itself, be taken as evidence against a genuine, MRI-detectable compositional abnormality; regional variation in baseline T1rho/T2 values in normal menisci is a further reminder that a single-region measurement may not represent the whole [26].

That repair and tear produced statistically indistinguishable elevation, both higher than degeneration, should be interpreted cautiously in a cross-sectional cohort. Meniscal repair capacity is largely restricted to the peripherally vascularised “red-red” and “red-white” zones, where healing proceeds through a vascular, scar-like response rather than regeneration of native architecture [1]—a plausible biological basis for repair tissue remaining quantitatively abnormal well beyond gross healing. Even tissue graded as successfully healed on second-look arthroscopy remains distinguishable from an intact meniscus on T2 mapping [9], a pattern corroborated at ultrahigh field strength in dedicated evaluation of meniscal repair tissue [27], consistent with the present finding across the postoperative intervals represented in this dataset.

Tear morphology showed numerical variation in endpoint values, but morphology subgroup counts were limited, and no statistically significant morphology effect was demonstrated. Morphology-specific interpretation should therefore remain exploratory in the present manuscript. In repaired lesions with a recorded postoperative interval, longer time since surgery was associated with a lower endpoint, though this cross-sectional, single-timepoint relationship should be interpreted as hypothesis-generating given the modest sample size (*n* = 40) and wide confidence interval. This direction is concordant with serial postoperative studies: synthetic MRI-derived T2 of repaired menisci declines across sequential timepoints and correlates strongly with a validated clinical outcome score [14], and a comparable decline follows arthroscopic reshaping of discoid meniscus [28]. Using defined 1-year MRI healing criteria, one study similarly shows a parallel reduction in tear-site signal and diastasis over a comparable interval [29], and early postoperative signal intensity carries prognostic value for longer-term outcomes after meniscal repair [30]. Longitudinal UTE-T2* data after ACL reconstruction show a similar pattern restricted to structurally intact menisci, with no equivalent decline in torn ones at reconstruction [10]. This nuance may explain why our cross-sectional study, drawn from lesions of mixed healing status, showed only a weak directionally consistent association compared with what a longitudinal serial design might detect. Qualitative MRI healing grade alone notably correlates only weakly with functional outcome after repair, reinforcing the potential value of a quantitative endpoint such as T2RV [31].

Expressing T2RV relative to internal-control tissue, rather than raw absolute values or an external normative population, is a methodological strength of this study, consistent with an analogous same-joint approach already formalised for cartilage repair tissue relative to adjacent healthy cartilage in the same patient [17], and with T2RV interpreted relative to a native-tissue reference being standard practice at this institution for postoperative cartilage graft assessment [18,19]. The present study extends this internal-reference strategy to meniscal tissue within a lesion-based cartilage MRI cohort.

These findings carry a potential clinical implication. Several studies suggest that meniscal tissue characterisation can carry prognostic weight. A landmark sham-controlled trial found no benefit of arthroscopic partial meniscectomy over sham surgery for degenerative meniscal tears without osteoarthritis [4], reinforcing the clinical stakes of separating structurally significant tears from less advanced degenerative meniscal change. Early repair of root-related tears is both clinically and economically superior to delayed diagnosis or meniscectomy [32,33], and greater postoperative meniscal extrusion is associated with downstream cartilage degeneration [34,35]. Further studies assessing symptoms, clinical decision-making, and functional outcomes are needed to determine whether T2RV can guide treatment selection or prognosis.

Our study has several limitations. First, this was a retrospective, single-centre study, and selection was based on incidental identification of pathology on a research-adjacent MRI protocol rather than a prospectively enrolled, consecutively imaged cohort. Second, requiring arthroscopic correlation as a uniform reference standard for inclusion, while resolving the earlier selective use of arthroscopy, introduced a new selection effect: repaired lesions and myxoid/intrasubstance degeneration were each identified around a surgical event and so retained arthroscopic correlation almost by definition, whereas most preoperatively identified tears in this cohort had not yet proceeded to surgery and were consequently excluded. The analysed tear subgroup (*n* = 12) is accordingly small and may be enriched for tears selected for surgery, which should be considered when interpreting the tear-versus-repair comparison. A related selection effect arising from excluding a small number of discordant cases (*n* = 11, 11% of the screened cohort) could be a limitation. This pattern likely reflects local imaging practice: at our institution, cartilage T2RV mapping is obtained primarily for postoperative surveillance of cartilage graft maturation, and less frequently for preoperative evaluation of knee pain in patients with normal-appearing radiographs; the T2RV sequence is not part of the routine knee MRI protocol and is only acquired when specifically requested by the referring clinician. Consequently, most meniscal tears that proceed to surgery are worked up with conventional MRI sequences alone, without a T2RV acquisition, and so never enter this cartilage-protocol cohort; the tears captured here instead represent the smaller subset incidentally identified on a T2RV-protocol scan performed for another indication. Third, the degeneration group remained comparatively small (*n* = 9 lesions), limiting the precision of its effect estimates. Fourth, T2RV was assessed at a single, cross-sectional timepoint per lesion; the postoperative-interval analysis in particular would benefit from prospective, serial imaging rather than a cross-sectional sample of differing intervals. Fifth, formal inter-reader reliability of ROI placement was not assessed, since placement and interpretation were performed by a single fellowship-trained musculoskeletal radiologist, and reproducibility across independent readers has not yet been assessed. Prior work applying T2 mapping to native and repair cartilage tissue has shown interobserver agreement can vary, particularly for repair tissue [36]. Finally, T2RV values were derived using a cartilage T2 mapping protocol rather than a sequence specifically optimised for meniscal imaging, and applicability of the findings to other MRI platforms or quantitative techniques remains to be established. These limitations notwithstanding, the internal consistency of the findings—a biologically graded elevation that remained significant after multivariable adjustment, together with a concordant postoperative trend—supports T2RV as a promising quantitative marker warranting prospective, multicenter validation.

## 5. Conclusions

In this retrospective cohort, an internal-control-normalised T2RV endpoint was elevated in pathologic meniscal tissue. Longer postoperative intervals were associated with a lower endpoint among repaired lesions in this cross-sectional analysis; this association should be interpreted as exploratory rather than as evidence of tissue maturation. These findings support internal-control-normalised T2RV as a non-invasive quantitative marker of meniscal tissue status, with potential to complement conventional MRI and clinical assessment, showing higher T2RV elevation in higher-grade structural pathology than in lower-grade intrasubstance degenerative change within this cohort. Prospective multicenter studies, blinded ROI placement, reader-reliability testing, and diagnostic-performance analyses are required before clinical adoption.

## Figures and Tables

**Table 1:** Patient demographics and lesion characteristics.

Demographic/Characteristic	Value
Patient demographics (*n* = 55 patients)	
Age (years), mean ± SD (range)	47.1 ± 8.8 (24–66)
Sex, *n* (%)	
Male	34 (61.8)
Female	21 (38.2)
Lesion characteristics (*n* = 61 lesions)	
Pathology group, *n* (%)	
Repair	40 (65.6)
Tear	12 (19.7)
Degeneration	9 (14.8)
Meniscus side, *n* (%)	
Medial	39 (63.9)
Lateral	22 (36.1)
Region, *n* (%)	
Posterior horn	27 (44.3)
Body	16 (26.2)
Anterior horn	12 (19.7)
Posterior root	6 (9.8)
Tear morphology ^1^, *n* (%) (*n* = 52 lesions)	
Complex	19 (36.5)
Radial	9 (17.3)
Horizontal	9 (17.3)
Vertical	7 (13.5)
Bucket-handle	5 (9.6)
Root-related	3 (5.8)
Surgery type ^2^, *n* (%) (*n* = 42 lesions)	
Repair	23 (54.8)
Debridement	9 (21.4)
Meniscectomy	8 (19.0)
Healed (no intraoperative repair required)	2 (4.8)
MRI indication, *n* (%)	
Post-operative	51 (83.6)
Pre-operative	10 (16.4)
Pathologic ROI T2RV (ms), mean ± SD	40.15 ± 8.89
Internal-control ROI T2RV (ms), mean ± SD	25.76 ± 0.56
Postoperative interval (days) ^3^, mean ± SD (range) (*n* = 40 repaired lesions)	250 ± 132 (140–846)
Postoperative interval, all pathology groups (days) ^4^, mean ± SD (range) (*n* = 51 postoperative scans)	256 ± 135 (140–846)
Preoperative interval (days) ^5^, mean ± SD (range) (*n* = 10 pre-operative scans)	387 ± 395 (32–1049)

^1^ Recorded for tear and repair lesions only; not applicable to myxoid/intrasubstance degeneration. ^2^ Recorded when a meniscal-specific intraoperative procedure or intraoperative finding was documented. ^3^ Time from surgery to MRI, calculated for repaired lesions with both a recorded surgery date and a postoperative MRI (*n* = 40); not calculable for lesions without both dates recorded. ^4^ As footnote 3, but calculated across all pathology groups with a postoperative MRI (*n* = 51: 9 degeneration, 40 repair, 2 tear). ^5^ Time from MRI to surgery, calculated for lesions with a preoperative MRI and a subsequently recorded surgery date (*n* = 10; all tear lesions). ROI, region of interest; SD, standard deviation; T2RV, T2 relaxation value.

**Table 2:** Primary analysis: pathologic versus internal-control ROI T2RV (*n* = 61 lesions).

Measure	Result
Pathologic ROI T2RV (ms), mean ± SD (median, IQR)	40.15 ± 8.89 (40.03, 32.44–45.11)
Internal-control ROI T2RV (ms), mean ± SD (median, IQR)	25.76 ± 0.56 (25.79, 25.30–26.16)
Paired difference (ms), mean ± SD (95% CI)	14.39 ± 8.70 (12.16 to 16.62)
Wilcoxon signed-rank test (primary inferential test)	*p* = 1.1 × 10^−11^
Normalised T2RV endpoint (%), mean ± SD (median, IQR)	55.7 ± 33.1 (56.2, 27.2–71.2)

CI, confidence interval; ROI, region of interest; SD, standard deviation; IQR, interquartile range; T2RV, T2 relaxation value.

**Table 3:** T2RV endpoint (%) by pathology group.

Pathology Group	Mean ± SD Endpoint (%)	Median Endpoint (%) (IQR)
Degeneration (*n* = 9)	17.5 ± 15.7	13.4 (10.8–16.1)
Repair (*n* = 40)	60.0 ± 26.1	57.3 (45.3–70.1)
Tear (*n* = 12)	69.9 ± 43.6	79.6 (24.8–100.3)

Kruskal–Wallis omnibus test: H = 14.79, *p* = 6.2 × 10^−4^. Dunn’s post hoc test with Holm correction for three comparisons: Degeneration vs. Repair *p* = 1.1 × 10^−3^; Degeneration vs. Tear *p* = 1.1 × 10^−3^; Repair vs. Tear *p* = 0.400. IQR, interquartile range; SD, standard deviation.

## Data Availability

The data that support the findings of this study are available from the corresponding author upon reasonable request, due to restrictions related to patient confidentiality and Institutional Review Board regulations.

## Supplementary Materials

The following supporting information can be downloaded at: https://www.mdpi.com/article/10.3390/diagnostics16162564/s1: Table S1: Sensitivity analysis: full lesion-level dataset versus one lesion per patient; Table S2: Parametric equivalent of the primary non-parametric test (paired t-test); Table S3: Within-group paired-test breakdown (pathologic vs. internal-control ROI T2RV) by pathology group; Table S4: Exploratory T2RV endpoint (%) comparisons by meniscal side, region, and tear morphology; Table S5: Multivariable linear regression of T2RV endpoint (%), with heteroscedasticity-robust (HC3) 95% confidence intervals and a patient-cluster-robust concordance check.

## Abbreviations

The following abbreviations are used in this manuscript:

T2RV	T2 relaxation value
ROI	Region of interest
MRI	Magnetic resonance imaging
ACL	Anterior cruciate ligament
FSE	Fast-spin echo
PD	Proton density
FS	Fat saturation
SD	Standard deviation
IQR	Interquartile range
CI	Confidence interval
IRB	Institutional Review Board
ARDL	AIR Recon DL
HC3	Heteroscedasticity-consistent (MacKinnon–White)
AI	Artificial intelligence
