# Supplementary material for: Internal-Control-Normalized T2 Mapping of Meniscal Abnormalities on a Cartilage MRI Protocol: An Exploratory Retrospective Study
Source: Diagnostics (Basel). 2026 Aug 14;16(16):2564. doi: 10.3390/diagnostics16162564 (PMC13512825; doi:10.3390/diagnostics16162564)
Supplement: Supplementary file 1 [file diagnostics-16-02564-s001.zip › diagnostics-4456564-supplementary.pdf]

MDPI diagnostics

Supplementary Materials

Internal-Control-Normalized T2 Mapping of Meniscal Abnormalities on a Cartilage MRI Protocol: An Exploratory Retrospective Study

This file provides supporting statistical detail referenced from the main manuscript: a sensitivity analysis restricted to one lesion per patient; a parametric equivalent of the primary non-parametric test; the full within-group paired-test breakdown by pathology group; full exploratory subgroup statistics; and heteroscedasticity- and patient-cluster-robust standard errors for the multivariable regression. None of the analyses in this file altered the principal conclusions reported in the main text (Sections 3.3 and 3.4).

Table S1. Sensitivity analysis: full lesion-level dataset versus one lesion per patient.

Analysis	Result
Pathology-group Kruskal–Wallis, arthroscopically correlated dataset (*n* = 61)	H = 14.79, $p = 6.2 \times 10^{-4}$
Pathology-group Kruskal–Wallis, one lesion/patient (*n* = 55)	H = 11.45, $p = 3.3 \times 10^{-3}$
Primary Wilcoxon signed-rank, arthroscopically correlated dataset (*n* = 61)	$p = 1.1 \times 10^{-11}$
Primary Wilcoxon signed-rank, one lesion/patient (*n* = 55)	$p = 1.1 \times 10^{-10}$

Restricting the dataset to one lesion per patient (retaining the first-recorded lesion for each of the 6 patients with multiple lesions) did not alter either primary conclusion.

Table S2. Parametric equivalent of the primary non-parametric test (paired t-test).

Analysis	Result
Primary analysis: paired t-test (pathologic vs. internal-control ROI T2RV, *n* = 61)	Mean paired difference 14.39 ± 8.70 ms (95% CI 12.16 to 16.62); $p = 5.5 \times 10^{-19}$

This parametric test was concordant with the primary non-parametric test reported in the main text (Wilcoxon signed-rank test), reported here as a robustness check. CI, confidence interval.

Table S3. Within-group paired-test breakdown (pathologic versus internal-control ROI T2RV) by pathology group.

Pathology group	Mean paired difference (ms), mean ± SD	Wilcoxon signed-rank test
Degeneration (*n* = 9)	4.55 ± 4.18	$p = 3.9 \times 10^{-3}$
Repair (*n* = 40)	15.46 ± 6.86	$p = 1.8 \times 10^{-12}$
Tear (*n* = 12)	18.23 ± 11.57	$p = 4.9 \times 10^{-4}$

Paired difference = pathologic ROI T2RV minus internal-control ROI T2RV, in ms. All lesions in every group showed a positive difference. SD, standard deviation.

Table S4. Exploratory T2RV endpoint (%) comparisons by meniscal side, region, and tear morphology.

Subgroup	T2RV endpoint (%)
**By meniscal side**	
Medial (*n* = 39), mean ± SD (median)	54.2 ± 33.3 (55.0)
Lateral (*n* = 22), mean ± SD (median)	58.4 ± 33.5 (56.6)

Kruskal–Wallis test	p = 0.730
**By region**	
Anterior horn (*n* = 12), median	53.7
Body (*n* = 16), median	65.7
Posterior horn (*n* = 27), median	39.9
Posterior root (*n* = 6), median	70.4
Kruskal–Wallis test	p = 0.050
**By tear morphology (tear and repair lesions, *n* = 52)**	
Bucket-handle (*n* = 5), median	69.8
Complex (*n* = 19), median	52.4
Horizontal (*n* = 9), median	56.2
Radial (*n* = 9), median	66.7
Root-related (*n* = 3), median	72.3
Vertical (*n* = 7), median	71.2
Kruskal–Wallis test	p = 0.139

SD, standard deviation. Small subgroups (e.g., posterior root, *n* = 6; root-related morphology, *n* = 3) limit statistical power for these comparisons. None of these comparisons reached statistical significance, although the regional comparison approached the threshold (p = 0.050).

Table S5. Multivariable linear regression of T2RV endpoint (%) (*n* = 61 lesions, 55 patients).

Term	Coefficient (β)	95% CI	P
Intercept	28.71	−42.84 to 100.26	0.424
Pathology group: Repair (ref. Degeneration)	37.54	19.74 to 55.35	9.5×10^{-5}
Pathology group: Tear (ref. Degeneration)	46.38	11.01 to 81.74	0.011
Sex: Male (ref. Female)	−1.32	−18.57 to 15.93	0.878
Meniscus: Medial (ref. Lateral)	3.66	−17.75 to 25.06	0.733
Region: Body (ref. Anterior horn)	3.81	−22.18 to 29.79	0.770
Region: Posterior horn (ref. Anterior horn)	−9.22	−32.86 to 14.43	0.438
Region: Posterior root (ref. Anterior horn)	6.52	−22.86 to 35.90	0.658
Age (years)	−0.12	−1.38 to 1.14	0.846

R^2 = 0.281; adjusted R^2 = 0.171. Coefficient, 95% confidence interval (CI), and P computed using HC3 (heteroscedasticity-consistent, MacKinnon–White) robust standard errors. Conclusions for the pathology group were unchanged using patient-cluster-robust standard errors accounting for six patients contributing >1 lesion (Repair P = 1.7×10^{-4}; Tear P = 6.4×10^{-3}). ref., reference category. Coefficients are unstandardized and expressed in percentage points of the T2RV endpoint.
